# A modeling study by response surface methodology and artificial neural network on culture parameters optimization for thermostable lipase production from a newly isolated thermophilic *Geobacillus *sp. strain ARM

**DOI:** 10.1186/1472-6750-8-96

**Published:** 2008-12-23

**Authors:** Afshin Ebrahimpour, Raja Noor Zaliha Raja Abd Rahman, Diana Hooi Ean Ch'ng, Mahiran Basri, Abu Bakar Salleh

**Affiliations:** 1 Faculty of Biotechnology and Biomolecular Sciences, Universiti Putra Malaysia, 43400 UPM Serdang, Selangor, Malaysia; 2 Faculty of Science, Universiti Putra Malaysia, 43400 UPM Serdang, Selangor, Malaysia

## Abstract

**Background:**

Thermostable bacterial lipases occupy a place of prominence among biocatalysts owing to their novel, multifold applications and resistance to high temperature and other operational conditions. The capability of lipases to catalyze a variety of novel reactions in both aqueous and nonaqueous media presents a fascinating field for research, creating interest to isolate novel lipase producers and optimize lipase production. The most important stages in a biological process are modeling and optimization to improve a system and increase the efficiency of the process without increasing the cost.

**Results:**

Different production media were tested for lipase production by a newly isolated thermophilic *Geobacillus *sp. strain ARM (DSM 21496 = NCIMB 41583). The maximum production was obtained in the presence of peptone and yeast extract as organic nitrogen sources, olive oil as carbon source and lipase production inducer, sodium and calcium as metal ions, and gum arabic as emulsifier and lipase production inducer. The best models for optimization of culture parameters were achieved by multilayer full feedforward incremental back propagation network and modified response surface model using backward elimination, where the optimum condition was: growth temperature (52.3°C), medium volume (50 ml), inoculum size (1%), agitation rate (static condition), incubation period (24 h) and initial pH (5.8). The experimental lipase activity was 0.47 Uml^-1 ^at optimum condition (4.7-fold increase), which compared well to the maximum predicted values by ANN (0.47 Uml^-1^) and RSM (0.476 Uml^-1^), whereas R^2 ^and AAD were determined as 0.989 and 0.059% for ANN, and 0.95 and 0.078% for RSM respectively.

**Conclusion:**

Lipase production is the result of a synergistic combination of effective parameters interactions. These parameters are in equilibrium and the change of one parameter can be compensated by changes of other parameters to give the same results. Though both RSM and ANN models provided good quality predictions in this study, yet the ANN showed a clear superiority over RSM for both data fitting and estimation capabilities. On the other hand, ANN has the disadvantage of requiring large amounts of training data in comparison with RSM. This problem was solved by using statistical experimental design, to reduce the number of experiments.

## Background

Today, lipases (EC 3.1.1.3, triacylglycerol acylhydrolases) stand amongst the most important biocatalysts. They carry out novel reactions in both aqueous and nonaqueous media. Lipases are used to hydrolyze ester bonds of a variety of nonpolar substrates at high activity, chemo-, region- and stereo-selectivity. Moreover, they are used to catalyze the reverse reactions (such as esterification [1] and transesterification [2]) in nonpolar solvents [3] and [4].

Among lipases of different sources, microbial thermostable lipases are highly advantageous for biotechnological applications, since they can be produced at low cost and exhibit improved stability [3]. Thus, various thermostable lipase-producing microorganisms have been isolated from diverse habitats [5-7].

Bacterial lipases are mostly extracellular and their production greatly influenced by nutritional and physico-chemical factors, such as nitrogen and carbon sources, metal ions, initial pH, temperature, medium volume, agitation rate, incubation period, inoculum size and aeration [8] and [9].

The most important stages in a biological process are modeling and optimization to improve a system and increase the efficiency of the process without increasing the cost [10]. The classical optimization method (single variable optimization) is not only time-consuming and tedious but also does not depict the complete effects of the parameters in the process and ignores the combined interactions between physicochemical parameters. This method can also lead to misinterpretation of results [10] and [11]. In contrast, response surface methodology (RSM) is an empirical modeling system for developing, improving, and optimizing of complex processes [12] and [5]. RSM assesses the relationships between the response(s) and the independent variables [13], and defines the effect of the independent variables, alone or in combination, in the processes.

Although RSM has so many advantages, and has successfully been applied to study and optimize the enzymatic processes [14] and [15], and enzyme production from microorganisms [16] and [17], it is hard to say that it is applicable to all optimization and modeling studies [18-20]. The past decade has seen a host of data analysis tools based on biological phenomena develop into well-established modeling techniques, such as artificial intelligence and evolutionary computing. Artificial neural networks (ANNs) are now the most popular artificial learning tool in biotechnology, with a wide applications range included optimization of bioprocesses [21] and enzyme production from microorganisms [22].

Indeed an ANN is a massively interconnected network structure consisting of many simple processing elements capable of performing parallel computation for data processing. The fundamental processing element of ANNs (the artificial neuron) simulates the basic functions of biological neurons [18] and [23].

In this work, after finding the best composition of production medium among the best previously published and modified media, the optimization of physical factors for extracellular thermostable lipase production from a newly isolated *Geobacillus *sp. strain ARM (DSM 21496 = NCIMB 41583) was carried out using RSM and ANN.

## Results and discussion

### Effect of various production media on lipase production

**Figure 1:**
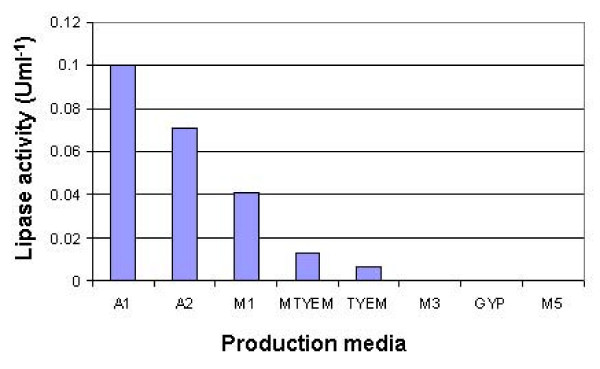
**Lipase activity in different compositions of production media**.

The production of lipases is mostly inducer-dependent [24] and different media have different stimulation effects on lipase production [9] based on the physiological and biochemical pathways of the bacterium. In order to select the best lipase production medium, the ability of bacterium to produce lipase was tested in eight different liquid media (Figure 1). Lipase activity in medium A1 was significantly higher than other production media, which is composed of peptone and yeast extract as organic nitrogen sources, olive oil as carbon source and lipase production inducer, sodium and calcium as metal ions, and gum arabic as emulsifier and lipase production inducer.

Generally, microorganisms provide high yields of lipase when organic nitrogen sources are used, such as peptone and yeast extract, which have been used for lipase production by various thermophilic *Bacillus *sp. [25,26] and [27]. Yeast extract is one of the most important nitrogen sources for high level lipase production by different microorganisms [28]. Besides this role, yeast extract supplies vitamins and trace elements for the growth of bacteria and increases their lipase production [29].

High levels of lipase production were reported from various thermophilic *Bacillus *sp. in the presence of olive oil as carbon source in the culture medium [6,27,30] and [28]. Most published experimental data have shown that lipid carbon sources (especially natural oils) stimulate lipase production [9,31] and [32], whereas carbon sources that are easily broken down and used by bacteria play an inhibitory role [30,33] and [34]. Different microorganisms have different requirement for metal ions. Calcium ions play essential roles for many microbial species. They are important in maintaining cell wall rigidity, stabilizing oligomeric proteins and covalently bounding protein peptidoglycan complexes in the outer membrane [35]. Lipase production by various *Bacillus *sp. was stimulated in the presence of Ca^2+ ^alone [26] and [36] or in combination with other ions such as Mg^2+^, and Fe^2+ ^[37].

On the other hand, highly branched, helically configurated, non-metabolizable polysaccharides such as gum arabic are able to enhance the lipase production. This might probably be due to the emulsification of culture media containing oil to increase the lipid surface (interfacial area between oil and water) for lipase action, detachment of lipase from the oil surface, and from binding sites at the outer membrane of Gram-negative bacteria [30,38] and [39].

As a result, A1 production medium was chosen as the medium to be used in the further optimization of lipase production.

### Analyzing and modeling

The central composite rotary design (CCRD) along with the observed responses is shown in Table 1.

**Table 1:** Experimental design used in RSM and ANN studies by using six independent variables showing observed values of lipase activity

**Temperature (°C)**	**Medium volume (ml)**	**Inoculum size (%)**	**Agitation rate (rpm)**	**Incubation period (h)**	**Initial pH**	**Lipase activity (Uml^-1^)**
60	162.5	4	50	60	6	0.2063

60	162.5	2	150	36	8	0

60	87.5	4	50	60	8	0.0207

50	162.5	2	150	60	8	0

60	87.5	4	150	36	8	0

60	162.5	2	50	60	8	0.0291

60	87.5	2	150	36	6	0

50	87.5	4	50	36	8	0.0886

50	162.5	2	50	36	8	0.0724

50	87.5	4	150	60	8	0

50	162.5	4	150	60	6	0.0907

50	87.5	2	50	36	6	0.1945

45	125	3	100	48	7	0.04

65	125	3	100	48	7	0.0229

55	50	3	100	48	7	0.0457

55	200	3	100	48	7	0.0551

55	125	1	100	48	7	0

55	125	5	100	48	7	0.0841

55	125	3	0	48	7	0.0675

55	125	3	200	48	7	0

55	125	3	100	24	7	0.0851

55	125	3	100	72	7	0.0281

55	125	3	100	48	5	0

55	125	3	100	48	9	0

*55*	*125*	*3*	*100*	*48*	*7*	*0.049*

*55*	*125*	*3*	*100*	*48*	*7*	*0.0475*

*55*	*125*	*3*	*100*	*48*	*7*	*0.046*

*55*	*125*	*3*	*100*	*48*	*7*	*0.0455*

*55*	*125*	*3*	*100*	*48*	*7*	*0.0445*

**60**	**162.5**	**4**	**150**	**36**	**6**	**0**

**50**	**162.5**	**4**	**50**	**36**	**6**	**0.0693**

**60**	**87.5**	**2**	**50**	**60**	**6**	**0.0792**

**50**	**87.5**	**2**	**150**	**60**	**6**	**0.0996**

ANN training set: normal and italic (center points) numbers

ANN testing set: bold numbers

### Response surface methodology

Fitting the data to various models (linear, two factorial, quadratic and cubic) and their subsequent ANOVA showed that all models were unable to explain the effects of physical factors on the lipase production. To overcome of this problem, we used backward elimination strategy followed by hierarchical terms addition to find the best model. Backward elimination started with all of the predictors in the model. The variable that was least significant (with the largest P-value) was removed and the model was refitted. Each subsequent step removed the least significant variable in the model until all remaining variables had individual P-values smaller than 0.05 [40]. Finally, modified cubic equation (equation 1) and its subsequent ANOVA (Table 2) showed a quite suitable model to optimize the lipase production. Indeed, the modified model was a quadratic model with one eliminated (V.Ag) and one additional (T.Ag.t) terms.

**Table 2:** ANOVA for joint test

**Source**	**Sum of Squares**	**DF**	**Mean Square**	**F Value**	**Prob > F**	
Model	0.083	*27*	3.075E-003	1176.88	< 0.0001	significant

*A*	*1.462E-004*	*1*	*1.462E-004*	*55.96*	*0.0007*	

*B*	*4.418E-005*	*1*	*4.418E-005*	*16.91*	*0.0092*	

*C*	*3.536E-003*	*1*	*3.536E-003*	*1353.65*	<*0.0001*	

*D*	*2.278E-003*	*1*	*2.278E-003*	*872.01*	<*0.0001*	

*E*	*1.625E-003*	*1*	*1.625E-003*	*621.82*	<*0.0001*	

*F*	*0.000*	*1*	*0.000*	*0.000*	*1.0000*	

*A*^2^	*3.236E-004*	*1*	*3.236E-004*	*123.86*	*0.0001*	

*B*^2^	*2.173E-005*	*1*	*2.173E-005*	*8.32*	*0.0344*	

*C*^2^	*2.829E-005*	*1*	*2.829E-005*	*10.83*	*0.0217*	

*D*^2^	*2.322E-004*	*1*	*2.322E-004*	*88.89*	*0.0002*	

*E*^2^	*1.457E-004*	*1*	*1.457E-004*	*55.78*	*0.0007*	

*F*^2^	*3.089E-003*	*1*	*3.089E-003*	*1182.37*	<*0.0001*	

*AB*	*5.105E-003*	*1*	*5.105E-003*	*1954.11*	<*0.0001*	

*AC*	*3.499E-003*	*1*	*3.499E-003*	*1339.22*	<*0.0001*	

*AD*	*2.252E-003*	*1*	*2.252E-003*	*862.12*	<*0.0001*	

*AE*	*1.873E-003*	*1*	*1.873E-003*	*716.75*	<*0.0001*	

*AF*	*2.059E-004*	*1*	*2.059E-004*	*78.82*	*0.0003*	

*BC*	*5.826E-003*	*1*	*5.826E-003*	*2229.90*	<*0.0001*	

*BE*	*4.489E-003*	*1*	*4.489E-003*	*1718.28*	<*0.0001*	

*BF*	*2.346E-003*	*1*	*2.346E-003*	*898.14*	<*0.0001*	

*CD*	*2.162E-005*	*1*	*2.162E-005*	*8.28*	*0.0347*	

*CE*	*2.992E-003*	*1*	*2.992E-003*	*1145.30*	<*0.0001*	

*CF*	*5.720E-005*	*1*	*5.720E-005*	*21.90*	*0.0054*	

*DE*	*9.310E-004*	*1*	*9.310E-004*	*356.38*	*< 0.0001*	

*DF*	*1.373E-003*	*1*	*1.373E-003*	*525.44*	*< 0.0001*	

*EF*	*6.529E-003*	*1*	*6.529E-003*	*2499.00*	*< 0.0001*	

*ADE*	*2.820E-003*	*1*	*2.820E-003*	*1079.53*	*< 0.0001*	

*Residual*	1.306E-005	*5*	2.613E-006			

*Lack of Fit*	*5.625E-007*	*1*	*5.625E-007*	*0.18*	*0.6932*	*not significant*

*Pure Error*	*1.250E-005*	*4*	*3.125E-006*			

Cor Total	0.083	32				

A: Temperature

B: Medium volume

C: Inoculum size

D: Agitation rate

E: Incubation period

F: Initial medium pH

(1) **Lipase activity (U ml^-1^) **= 4.41 - 0.06 T - 0.01 V - 0.32 IS - 0.02 Ag - 0.07 t + 0.11 pH - 1.5E-4 T^2 ^+ 6.9E-7 V^2 ^- 1.1E-3 IS^2 ^- 1.3E-6 Ag^2 ^+ 1.7E-5 t^2 ^- 0.01 pH^2 ^+ 9.5E-5 T.V + 3E-3 T.IS + 3.8E-4 T.Ag + 1.3E-3 T.t + 7.2E-4 T.pH + 8.8E-4 V.IS + 3.7E-5 V.t + 5.6E-4 V.pH - 2.3E-5 IS.Ag + 1.1E-3 IS.t + 3.3E-3 IS.pH + 5.5E-4 Ag.t + 1.8E-4 Ag.pH - 1.7E-3 t.pH - 9.7E-6 T.Ag.t

where T is temperature, V is medium volume, IS is inoculum size, Ag is agitation rate, t is incubation period and pH is initial medium pH.

The computed model F-value of 1176.88 implies the model is significant and there is only a 0.01% chance that a "model F-value" this large could occur due to noise. The 'lack of fit F-value" of 0.18 implies the lack of fit is not significant relative to the pure error. There is a 69.32% chance that a "lack of fit F-value" this large could occur due to noise. Non-significant lack of fit shows the model is significant. On the other hand, the pure error is very low, indicating good reproducibility of the data obtained. With very small "model P-value" (< 0.0001) and large "lack of fit P-value" (0.6932) from the analysis of ANOVA and a suitable coefficient of determination (R^2 ^= 0.9998) and adjusted coefficient of determination (R^2^_adjusted _= 0.999), the modified cubic polynomial model was highly significant and sufficient to represent the actual relationship between the response and the significant variables (Table 2).

### Artificial neural network

#### Effect of architecture and topology on neural network performance

The selection of an optimal neural-network architecture and topology is of critical importance for a successful application. Several neural-network architectures and topologies were tested for the estimation and prediction of lipase production. Table 3 summarizes the top five ANN models.

**Table 3:** The effect of different neural network architecture and topologies on coefficient of determination, *R*^2^, and absolute average deviation, AAD, in the estimation of lipase production obtained in the training and testing of neural networks

**Name**	**Model**	**Learning algorithm**	**Connection type**	**Transfer function output**	**Transfer function hidden**	**Training set R**^2^	**Training set AAD (%)**	**Testing set R**^2^	**Testing set AAD (%)**
C21	4-16-1	IBP^a^	MFFF^b^	Linear	Gaussian	1	0.1	1	0.231

D25	4-16-1	IBP	MNFF^c^	Linear	Gaussian	1	0.145	0.99	0.358

C12	4-15-1	IBP	MFFF	Linear	Gaussian	1	0.138	0.953	0.455

J22	4-15-1	IBP	MNFF	Linear	Tanh^d^	1	0.167	0.938	0.552

H5	4-15-1	IBP	MFFF	Linear	Tanh	1	0.196	0.908	0.639

^a ^Incremental Back Propagation

^b ^Multilayer Full FeedForward

^c ^Multilayer Normal FeedForward

^d ^Hyperbolic Tangent Function

#### Effect of learning algorithm and transfer function

Training a neural network model essentially means selecting one model from the set of allowed models that minimizes the cost criterion. We have tested different learning algorithms for training neural network models. All accepted models (RMSE < 0.0001, R = 1 and DC = 1) have shown that incremental back propagation (IBP) was the most suitable learning algorithm for prediction of lipase production (Table 3).

The type of transfer function employed affects the neural network's learning rate and is instrumental in its performance. In the present work, among all employed transfer functions for hidden and output layers, accepted models were produced by linear function for output layer and Gaussian function or hyperbolic tangent (Tanh) for hidden layer that between them, the best models have been obtained by Gaussian function.

#### Optimal number of hidden neurons

**Figure 2:**
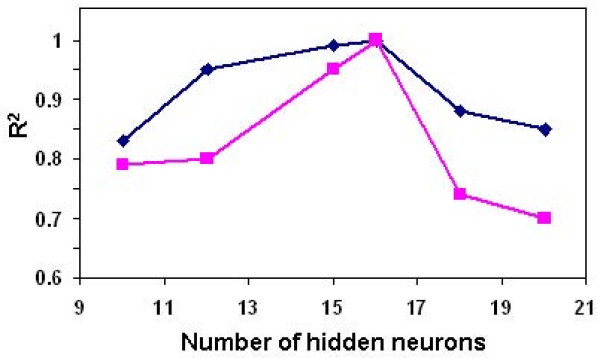
**Optimal number of hidden neurons**. Estimation of lipase production with neural networks of varying number of hidden neurons, tested with two example cases: incremental back propagation multilayer full feedforward (blue diamond) and multilayer normal feedforward incremental back propagation (pink square) with Gaussian transfer functions.

Although it is important to select the optimal number of hidden neurons carefully, depending on the type and complexity of the task, this usually has to be done by trial and error. An increase in the number of hidden neurons up to a point usually results in a better learning performance. Too few hidden neurons limit the ability of the neural network to model the process, and too many may allow too much freedom for the weights to adjust and, thus, to result in learning the noise present in the database used in training [41]. We tested the effect of number of hidden neurons on the goodness of fit. The results of testing with the two sample experiments, evaluated statistically on the basis of the coefficient of determination (R^2^), are shown in Figure 2. In both examined cases, the optimum number of hidden neurons was 16, with an obvious overfitting when too many hidden neurons were used. Then the 6-16-1 topology was chosen as the best topology for estimation of lipase production.

#### Artificial neural network analysis of lipase production

The best ANN chosen in the present work was a multilayer full feedforward incremental back propagation network with Gaussian transfer function (Table 3, C21) that consisted of a 6-16-1 topology. The optimized values of network for learning rate and momentum were 0.15 and 0.8, respectively. The learning was completed in RMSE = 9.99E-5, R = 1 and DC = 1. In the case of training data set, the coefficient of determination (R^2^) and absolute average deviation (AAD) were 1 and 0.1%, respectively, whereas for the testing data set, R^2 ^was 1 and AAD was 0.231% (Table 4) and for validating data sets R^2 ^and AAD were 0.989 and 0.059%, respectively (Table 5). Comparison of predicted and experimental values in training, testing and validating data sets, not only revealed capability of ANN in prediction of known data responses (the data that have been used for training) but also showed the ability of generalization for unknown data (the data that have not been used for training) and implying that empirical models derived from ANN can be used to adequately describe the relationship between the input factors and lipase production.

**Table 4:** Actual and predicted lipase activity by ANN and RSM models along with absolute deviation, R^2 ^and AAD

**Actual activity**	**ANN predicted activity**	**ANN absolute deviation**	**RSM predicted activity**	**RSM absolute deviation**
0.2063	0.2063	0	0.2061	0.000969

0	0	0	0.00019	0

0.0207	0.0208	0.0048	0.0209	0.0097

0	0.0001	0	0.0002	0

0	0.0002	0	0.0002	0

0.0291	0.0291	0	0.0289	0.0069

0	0	0	0.0002	0

0.0886	0.0886	0	0.0888	0.0023

0.0724	0.0724	0	0.0722	0.0028

0	0	0	0.0002	0

0.0907	0.0908	0.0011	0.0909	0.0022

0.1945	0.1945	0	0.1947	0.001

0.04	0.04	0	0.04	0

0.0229	0.0227	0.0087	0.0229	0

0.0457	0.0457	0	0.0457	0

0.0551	0.0552	0.0018	0.0551	0

0	0	0	0	0

0.0841	0.084	0.0012	0.0841	0

0.0675	0.0677	0.003	0.0675	0

0	0	0	0	0

0.0851	0.0851	0	0.0851	0

0.0281	0.028	0.0044	0.0281	0

0	0	0	0	0

0	0	0	0	0

*0.0467*	*0.0467*	*0*	*0.0465*	*0.0043*

**0**	**0.001**	0	0.0002	0

**0.0693**	**0.0692**	0.0014	0.0691	0.0029

**0.0792**	**0.0795**	0.0038	0.0794	0.0025

**0.0996**	**0.0992**	0.004	0.0994	0.002

ANN training set R^2 ^= 1

ANN training set AAD = 0.1%

RSM R^2 ^= 1

RSM AAD = 0.129%

ANN testing set R^2 ^= 1

ANN testing set AAD = 0.23117%

ANN training set: normal and italic (center points) numbers

ANN testing set: bold numbers

**Table 5:** Solution of optimum condition

**No.**	**Growth temperature (°C)**	**Medium volume (ml)**	**Inoculum size (%)**	**Agitation rate (rpm)**	**Incubation period (h)**	**Initial pH**	**ANN predicted activity (Uml^-1^)**	**Actual activity ± SD (Uml^-1^)**	**RSM predicted activity (Uml^-1^)**
1	52.3	50	1	0	24	5.8	0.47	0.47 ± 0.003	0.476

2	50	50	2	10	24	6	0.453	0.453 ± 0.002	0.458

3	48	50	1.4	0	33.6	5.8	0.44	0.445 ± 0.005	0.444

4	48	50	1.4	10	33.6	5.7	0.424	0.425 ± 0.003	0.43

5	48	50	1.4	20	33.6	5.8	0.423	0.423 ± 0.006	0.423

6	49	50	1	40	33.6	6	0.418	0.419 ± 0.002	0.411

7	48	50	1.5	20	33.6	5.8	0.412	0.413 ± 0.001	0.415

ANN R^2 ^= 0.989

ANN AAD = 0.059%

RSM R^2 ^= 0.95

RSM AAD = 0.078%

#### Comparison of RSM and ANN predicted values

The predicted output values of RSM and ANN are shown in Table 4. Though both models preformed well and offered stable responses, yet the ANN based approach was better in both data fitting and estimation capabilities in comparison to the RSM.

#### Main effects and interaction between parameters

The optimum level of each variable and the effect of their interactions on lipase production as a function of two variables were studied by plotting three dimensional response surface curves (while keeping the other variables at optimum point).

**Figure 3:**
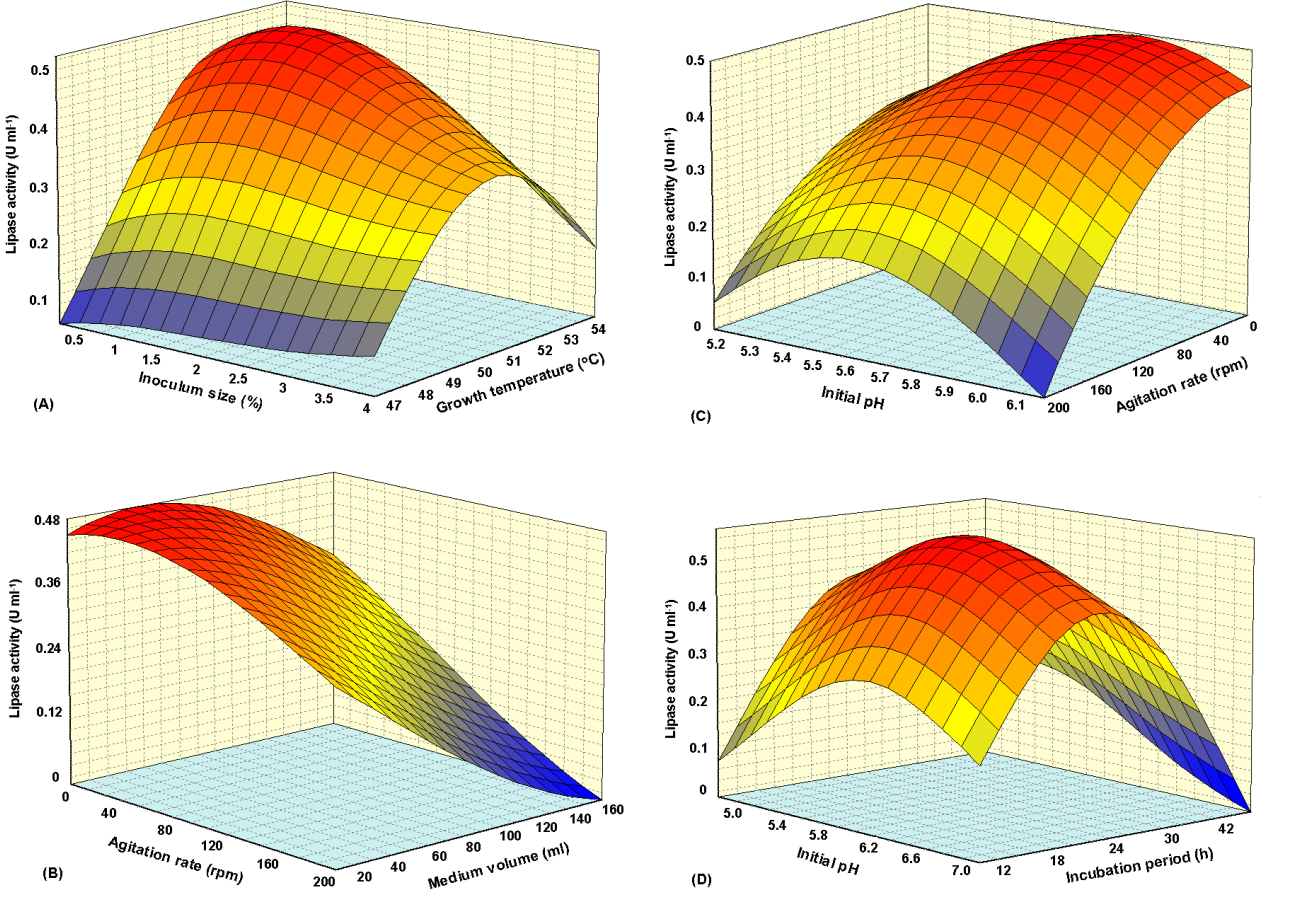
**Three dimensional plot showing the effect of: (A) growth temperature, inoculum size; (B) agitation rate, medium volume; (C) initial pH, agitation rate; and (D) initial pH, incubation period, and their mutual effect on the lipase production**. Other variables are constant: growth temperature (52.3°C), medium volume (50 ml), inoculum size (1%), agitation rate (static condition), incubation period (24 h) and initial pH (5.8).

ANOVA analysis (Table 2) and three dimensional plots (Figure 3) reveal that growth temperature, medium volume, inoculum size and incubation period had significant effects on lipase production. ANOVA analysis shows that although pH was not a significant parameter (P value > 0.05), it had important and significant interactions with other parameters, hence it has been used to develop the model. On the other hand, among the different interactions, interaction between agitation rate and growth volume, did not show significant effect on lipase production (P value > 0.05). Figure 3B depicts that lipase activity effectively increased with a decrease in growth volume but agitation rate did not show significant effect on lipase production. On the other hand, ANOVA analysis and Figure 3D reveal, that agitation is one of the most important parameters for lipase production. As a conclusion, though both agitation rate and growth volume parameters are significant, yet their interaction is not a significant parameter for lipase production. Hence modification of model via the removal of this interaction using backward elimination strategy improved the model (Equation 1).

Figure 3A represents the three dimensional plot as function of temperature and inoculum size on lipase activity. Maximum lipase activity of 0.47 Uml^-1 ^was obtained at the 52.3°C and 1.0% inoculum size. Further increase or decrease in the temperature, and increase in the inoculum size led to the decrease in the enzyme production. Generally, the optimum temperature for lipase production corresponds with the growth temperature of the respective microorganism [26]. It has been also proven that temperature regulate enzyme synthesis at mRNA transcription and probably translation levels. For extracellular enzymes, temperature influences their secretion, possibly by changing the physical properties of the cell membrane [42]. On the other hand, though higher temperature causes higher reaction rates and higher solubility of substrate and products, yet oxygen solubility is usually decreased.

At a suitable inoculum size, the nutrient and oxygen levels are enough for sufficient growth of bacteria and therefore, enhance the lipase production. If the inoculum size is too small, insufficient number of bacteria will lead to reduced amount of secreted lipase. High inoculum size can result in the lack of oxygen and nutrient depletion in the culture media [42] and [43].

Figures 3B and 3C depict the medium volume-agitation rate and initial pH-agitation rate interactions respectively. These plots reveal that the lipase activity increased with a decrease in culture volume and agitation rate. The maximum lipase activity was obtained at 50 ml culture volume and moderately acidic pH (5.8), under static condition. Similarly, static condition had resulted in comparatively high lipase production for *Syncephalastrum racemosum *[44]*Pseudomonas *sp. strain S5 [45] and *Pseudomonas aeruginosa *[46]. Generally, suitable agitation lead to sufficient supply of dissolved oxygen in the media [47]. Nutrient uptake by bacteria also will be increased [48], but the degree of aeration appears to be critical in some cases since shallow layer (static) cultures (moderate aeration) produced much more lipase than shake cultures (high aeration) [45].

The medium volume may have a great effect on the enzyme production. Although a larger medium volume initially contains more oxygen, nutrients and space for growth of bacteria, the void in the container and subsequently oxygenation of the medium will be decreased. On the other hand, it seems that ratio of surface area to volume (A/V) is important for lipase production where higher ratio cause higher oxygenation and lipase production [49].

The combined effect of initial pH and incubation time on lipase production is shown in Figure 3D. According to the plot, a moderately acidic initial pH (5.8) caused maximum lipase production after 24 h of cultivation. The activity was decreased remarkably as the incubation period changed. pH plays an important role in all the biological processes. The initial pH of the growth medium is important for lipase production [8]. Most bacteria prefer neutral initial pH for the best growth and lipase production, such as thermophilic *Bacillus *sp. strains L2 and 398 [50] and [51]. Maximum lipase activity at higher initial pH by various thermophilic *Bacillus *sp. has also been reported [25] and [32]. In contrast, Ertugrul *et al*. [52] have reported a moderately acidic pH (6.0) as the optimum initial pH for lipase production by *Bacillus *sp. The molecular electric charges and consequently molecular interactions and functions are directly related to media pH, thus any changes in medium pH affects many biological functions such as enzymatic processes, signaling pathways and transportations of various components across the cytoplasmic membrane and cell wall [53]. Therefore, medium pH is very important in nutrients absorption and growth of bacteria, stimulation of enzyme production via signaling pathways and release of extracellular enzymes (based on proteolytic mechanism of signal peptidases that has been explained by Paetzel *et al*. [54]).

Lipases are produced throughout bacterial growth, with peak production being obtained by late exponential growth phase [55]. Therefore, the optimum incubation time is based on duration of log phase that is influenced by environmental conditions as well as by characteristics of the organism itself.

Different optimum conditions for maximum lipase production by various thermophilic *Bacillus *sp. were reported [25,32,50] and [51]. Strain differences and synergistic effects with other factors present in the medium might be responsible for differences in the obtained results. Although no conclusive picture has been emerged so far from the large amount of experimental data concerning the physiology of lipase biosynthesis and its regulation, most of published experimental data seem to support the following inference. At the end of log phase, when one of the essential nutrients of the culture medium is used up or some waste product of organism builds up in the medium to an inhibitory level, microorganisms try to solve the problem and continue the growth. One response to this problem is the production of extracellular hydrolytic enzymes such as lipases, proteases and amylases. In other words, limitation of growth can be an inducer for the production of some enzymes. On the other hand, Table 6 shows that lipase production is the result of a synergistic combination of effective parameters interactions. These parameters are in equilibrium. It means that a change of one parameter can be compensated by changes of other parameters to give same results.

**Table 6:** Effect of different combinations of parameters on lipase production

**No.**	**Temperature (°C)**	**Volume (ml)**	**Inoculum Size (%)**	**Agitation (rpm)**	**Time (h)**	**pH**	**Activity (U ml^-1^)**
1	63	200	4.6	60	43.2	5.4	**0.15**

2	47	200	2.2	20	24	7.4	**0.15**

2	53	200	4.2	80	57.6	7	**0.15**

3	59	65	3.8	100	24	7	**0.15**

5	45	50	1.8	160	48	6.2	**0.2**

6	47	80	1.8	180	28.8	6.6	**0.2**

7	61	50	2.6	60	33.6	5	**0.2**

8	57	125	3.4	0	72	5.8	**0.2**

9	47	95	1	20	38.4	6.6	**0.3**

10	49	50	4.6	0	38.4	5.4	**0.3**

11	53	80	2.2	100	24	6.2	**0.3**

12	55	50	3.4	60	28.8	6.6	**0.3**

**Figure 4:**
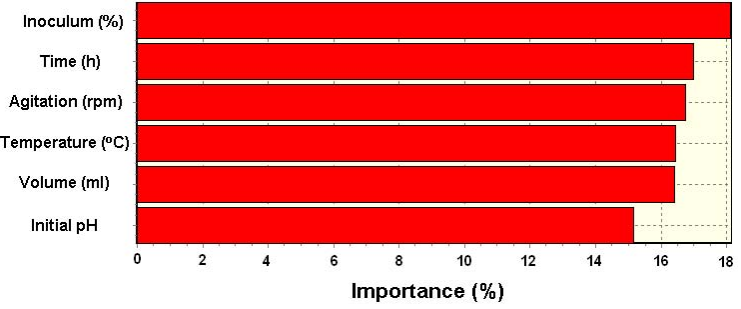
**Importance of effective parameters on lipase production**.

Finally, Figure 4 shows the importance percentage of effective parameters on the lipase production. Inoculum size of 18.15% is the most important factor on the lipase production, incubation period of 17.01%, agitation rate of 16.78%, growth temperature and medium volume of 16.46% and 16.44% respectively, and pH of 15.19% are subsequent degrees of importance.

### Optimization of reaction

The optimal conditions for lipase production were predicted as presented in Table 5 along with their predicted and actual values. Among the various optimum conditions, the highest lipase activity (0.47 Uml^-1^; 4.7-fold increase) was obtained at following conditions, growth temperature (52.3°C), medium volume (50 ml), inoculum size (1%), agitation rate (static condition), incubation period (24 h) and initial pH (5.8). Attention to R^2 ^and AAD values between actual and estimated responses demonstrated a higher prediction accuracy of ANN compared to RSM.

## Conclusion

In this work, different production media were tested for lipase production by a newly isolated thermophilic *Geobacillus *sp. strain ARM (DSM 21496 = NCIMB 41583). The maximum production was obtained in presence of peptone and yeast extract as organic nitrogen sources, olive oil as carbon source and lipase production inducer, sodium and calcium as metal ions, and gum arabic as emulsifier and lipase production inducer. On the other hand, culture parameters optimization and estimation of lipase production using RSM and ANN methods were successfully carried out. The best models were achieved by multilayer full feedforward incremental back propagation network and modified response surface model using backward elimination, where the optimum condition was: growth temperature (52.3°C), medium volume (50 ml), inoculum size (1%), agitation rate (static condition), incubation period (24 h) and initial pH (5.8). The experimental lipase activity was 0.47 Uml^-1 ^at optimum condition (4.7-fold increase), which compared well to maximum predicted values by ANN (0.47 Uml^-1^) and RSM (0.476 Uml^-1^), whereas R^2 ^and AAD were determined as 0.989 and 0.059% for ANN, and 0.95 and 0.078% for RSM respectively. Though the modified response surface model was comparable to ANN to provide good quality predictions for the six independent variables in terms of the lipase production, yet the ANN showed a clear superiority over RSM as a modeling technique for data sets showing nonlinear relationships.

On the other hand, ANN has the disadvantage of requiring large amounts of training data in comparison with RSM [56]. This problem was solved by using statistical experimental design, to reduce the number of experiments. Some of other researchers also have employed this strategy. Manohar and Divakar [21] employed a five variable parametric study for ANN analysis. They used 13 different combinations for training of network. Central composite design (CCD) was used for extracellular protease production (14 different combinations) [22] and modeling the growth of a bacterium (25 different combinations) [60]. Bas and Boyaci [18] employed face-centered design (FCD) and modified face-centered design (MFCD) for ANN study (13 different combinations for training).

As a conclusion lipase production is the result of a synergistic combination of effective parameters interactions. These parameters are in equilibrium and the change of one parameter can be compensated by changes of other parameters to give the same results. In addition, ANN can be a very powerful and flexible tool for modeling of the optimization process.

## Methods

### Bacterial strain

The bacterial strain used in this study was isolated from contaminated soil with oil from Selangor, Malaysia and identified as *Geobacillus *sp. strain ARM via 16S rDNA analysis [GenBank:EF025325] and deposited in DSMZ, Germany (DSM 21496) and NCIMB, UK (NCIMB 41583). This strain was preserved in sterile 16% (v/v) glycerol in Tryptic Soy Broth (TSB) at -80°C.

### Composition of lipase production medium

In order to select the best lipase production medium, eight different media were tested. The composition of the media was (% w/v): **M1**: peptone (3), yeast extract (1), NaCl (0.5), olive oil (1% v/v) [57]; **A1 **(modified M1): M1+ CaCl_2_.2H2O (0.05) + gum arabic (1); **A2 **(modified M1): A1+ MgSO_4_.7H_2_O (0.01), FeCl_3_.6H_2_O (0.004); **GYP**: glucose (2), yeast extract (1), peptone (1), CH_3_COONa· 3H_2_O (1), MgSO_4_·7H_2_O (0.03), MnSO_4 _(0.01), KCl (0.05), olive oil (2% v/v) [57]; **M3**: nutrient broth (0.325), gum arabic (1), CaCl_2_·2H_2_O (0.05), Tween 80 (1% v/v), olive oil (1% v/v) [57]; **M5**: nutrient broth (0.8), triolein (1% v/v) [23]; **TYEM**: tryptone (0.6), yeast extract (0.2), CaCl_2_.2H_2_O (0.02), MgSO_4_.7H_2_O (0.01), FeCl_3_.6H_2_O (0.04), olive oil (1.5% v/v) [30]; **MTYEM **(modified TYEM): tryptone (0.6), yeast extract (0.2), CaCl_2_.2H_2_O (0.02), MgSO_4_.7H_2_O (0.01), FeCl_3_.6H_2_O (0.04), gum arabic (1), olive oil (1.5% v/v).

The media were sterilized for 15 min at 121°C after pH adjustment to 7.0. Bacterial inoculum (2% v/v; Ab_600 _= 0.5 of overnight culture in TSB) was then inoculated into 50 ml production medium and incubated by agitation under 150 rpm, for 48 h at 60°C. The cell free supernatant was obtained by centrifugation at 12,000 *g*, 4°C for 15 min prior to lipase assay.

### Lipase activity assay

Determination of liberated free fatty acid was measured by colorimetric assay [58] using olive oil as substrate. The enzymatic reaction was performed in a water bath shaker for 30 min at 50°C under 200 rpm agitation. One unit of lipase activity was defined as 1.0 μmol of free fatty acid liberated min^-1 ^and reported as Uml^-1^.

### Experimental design

A five-level-six-factor central composite rotary design (CCRD) was employed in this study, requiring 33 experiments [59]. The variables and their levels selected for the lipase production optimization were: growth temperature (45 – 65°C); medium volume (50 – 200 ml); inoculum size (1 – 5%); agitation rate (0 – 200 rpm); incubation period (24 – 72 h) and initial pH (5 – 9). The experimental data [40 points include CCRD design (Table 1) and optimization data (Table 5)] was divided into three sets: training set, testing set and validating set. All tests were performed in triplicate.

### Response surface methodology analysis

The CCRD design experimental data was used for model fitting in RSM to find the best polynomial equation. This data was analyzed using Design Expert version 6.06 (Stat Ease Inc. Minneapolis, USA) and then interpreted. Three main analytical steps: analysis of variance (ANOVA), a regression analysis and the plotting of response surface were performed to establish an optimum condition for the lipase production. Then, the predicted values obtained from RSM model, were compared with actual values for testing the model. Finally, the experimental values of predicted optimal conditions (Table 5) were used as validating set and were compared with predicted values.

### Artificial neural network analysis

A commercial ANN software, NeuralPower version 2.5 (CPC-X Software) was used throughout the study. Multilayer normal feedforward and multilayer full feedforward neural networks were used to predict the lipase activity. Networks were trained by different learning algorithms (incremental back propagation, IBP; batch back propagation, BBP; quickprob, QP; genetic algorithm, GA; and Levenberg-Marquardt algorithm, LM). The ANN architecture consisted of an input layer with six neurons, an output layer with one neuron, and a hidden layer. To determine the optimal network topology, only one hidden layer was used and the number of neurons in this layer and the transfer functions of hidden and output layers (sigmoid, hyperbolic tangent function, Gaussian, linear, threshold linear and bipolar linear) were iteratively determined by developing several networks. Each ANN was trained until the network root of mean square error (RMSE) was lower than 0.0001, average correlation coefficient (R) and average determination coefficient (DC) were equal to 1. Other ANN parameters were chosen as the default values of the software. In the beginning, weights were initialized with random values and adjusted through a training process in order to minimize network error.

The CCRD design experimental data was divided into training and testing sets. For training, 25 points were used (Tables 1 and 4). One strategy for finding the best model is to summarize the data, it is well established that in ANN modeling, the replicates at center point do not improve the prediction capability of the network because of the similar inputs [10]. Hence, we improved our model by using mean of center points instead of 5 center points (Tables 1 and 4, italic numbers). To test the network, 4 remaining points were used (Tables 1 and 4, bold numbers). On the other hand, experimental values of predicted optimal conditions (Table 5) were used as validating set.

### Verification of estimated data

To test the estimation capabilities of the techniques, the estimated responses obtained from RSM and ANNs were compared with the observed responses. The coefficient of determination (R^2^) and absolute average deviation (AAD) were determined and these values were used together to compare ANNs to each other for finding the best ANN model, and the best ANN model with RSM. The AAD and R^2 ^are calculated by equations 2 and 3, respectively.

(2) $$\text{AAD}=\left\{\left[\sum_{\text{i}=1}^{\text{p}}\left(\left|{\text{y}}_{\text{i},\exp}-{\text{y}}_{\text{i},\text{cal}}\right|/{\text{y}}_{\text{i},\exp}\right)\right]/\text{p}\right\}\times 100$$

where *y*_*i*,exp _and *y*_*i*,cal _are the experimental and calculated responses, respectively, and *p *is the number of the experimental run.

(3) $${\text{R}}^{2}=1-\frac{\frac{\Sigma }{\text{i}=1\text{-n}}{\left({\text{model prediction}}_{\text{i}}-{\text{experimental value}}_{\text{i}}\right)}^{2}}{\frac{\Sigma }{\text{i}=1\text{-n}}{\left(\text{average experimental value}-{\text{experimental value}}_{\text{i}}\right)}^{2}}$$

where *n *is the number of experimental data.

R^2 ^is a measure of the amount of the reduction in the variability of response obtained by using the repressor variables in the model. Because R^2 ^alone is not a measure of the model's accuracy, it is necessary to use absolute average deviation (AAD) analysis, which is a direct method for describing the deviations. Evaluation of R^2 ^and AAD values together would be better to check the accuracy of the model. R^2 ^must be close to 1.0 and the AAD between the predicted and observed data must be as small as possible. The acceptable values of R^2 ^and AAD values mean that the model equation defines the true behavior of the system and it can be used for interpolation in the experimental domain [10].

## Authors' contributions

ABS, RNZRAR and MB conceived the idea of the study and experimental design. AE and DCHE performed the experiments described in this paper. AE conceived the RSM and ANN design and analysis, compared the estimation capabilities of the RSM with ANN and drafted the manuscript. All authors read and approved the final manuscript.
